# Toxocariasis in immigrants and travelers with unexplained eosinophilia

**DOI:** 10.1186/s13071-026-07300-9

**Published:** 2026-02-20

**Authors:** Laura Niño-Puerto, Belén Vicente, Josué Pendones Ulerio, Hugo Almeida, Javier Pardo Lledías, Juan Luis Muñoz Bellido, Antonio Muro, Moncef Belhassen-García

**Affiliations:** 1 Grupo Enfermedades Infecciosas y Tropicales (E-INTRO), Instituto de Investigación Biomédica de Salamanca (IBSAL), Centro de Investigación de Enfermedades Tropicales de la Universidad de Salamanca (CIETUS), Faculty of Pharmacy, Complejo Asistencial Universitario de Salamanca (CAUSA), University of Salamanca, Salamanca, Spain; 2 Microbiology and Parasitology Service, CAUSA, IBSAL, CIETUS, University of Salamanca, Salamanca, Spain; 3 Internal Medicine Service, Infectious Diseases Unit. E-INTRO, CAUSA, IBSAL, CIETUS, University of Salamanca, Salamanca, Spain; 4 Internal Medicine Service, Hospital Marqués de Valdecilla, University of Cantabria, Instituto de Investigación Valdecilla (IDIVAL), Santander, Spain

**Keywords:** Eosinophilia, *Toxocara canis*, ELISA, Spain, Western blot

## Abstract

**Background:**

Eosinophilia is common in immigrants and travelers and is often linked to parasitic infections. While well-known helminths are routinely considered, *Toxocara* spp. remains underrecognized despite its global prevalence. This study aimed to identify undiagnosed *T. canis* infections in migrant and traveler patients from tropical and subtropical regions with eosinophilia of unknown etiology.

**Methods:**

We retrospectively analyzed patients evaluated at the Tropical Medicine Unit of the Complejo Asistencial Universitario de Salamanca between 2008 and 2023. Eligible participants were immigrants or travelers from tropical and subtropical regions with eosinophilia and complete clinical records. Noninfectious causes were excluded before testing. Serum samples from patients without a confirmed parasitic diagnosis were screened for anti-*T. canis* immunoglobulin (Ig)G antibodies via enzyme-linked immunosorbent assay (ELISA), and positive results were confirmed via western blot. Demographic, clinical, and laboratory data were collected, and univariate analyses were used to assess associations with *T. canis* seropositivity.

**Results:**

Of the 192 patients tested, 44 (23.0%) were positive by ELISA, and 41 of 192 patients were confirmed by Western blot, the three discordant cases were considered indeterminate. Most were immigrants (37 of 41; 90%) from Africa (21 of 41; 51.2%) and Latin America (20 of 41; 48.8%), with a mean age of 28 years. Absolute eosinophilia was present in 70.5% of the positive patients, with a median eosinophil count of 946.4 cells/μL and a median total IgE level of 473 U/mL. Common symptoms included gastrointestinal complaints (41.7%), pruritus (37.5%), and fever (12.5%), while 40.9% were asymptomatic. Eosinophilia < 1000 cells/μL was significantly associated with seropositivity (OR 3.2, 95% CI 1.3–7.6; *P* = 0.009).

**Conclusions:**

*T. canis* infection is an important and frequently underrecognized cause of eosinophilia in immigrants and travelers. The systematic inclusion of *Toxocara* serology in the workup of unexplained eosinophilia, after ruling out common etiologies, may improve detection and guide management.

**Supplementary Information:**

The online version contains supplementary material available at 10.1186/s13071-026-07300-9.

## Background

Eosinophilia, which is commonly defined as an absolute eosinophil count above 450 cells/μL, is a significant clinical marker that may indicate a wide range of underlying conditions, including allergic, infectious, autoimmune, and neoplastic disorders [1]. Among immigrants and travelers from tropical and subtropical regions, helminthic infections are the leading cause, most frequently involving *Strongyloides stercoralis*, *Schistosoma* spp., hookworms, and other soil-transmitted helminths [2–6]. However, even after comprehensive evaluations with stool examinations and serological testing for common parasites, a substantial proportion of patients (up to 40–50%) remain with unexplained eosinophilia, often labeled idiopathic or of unknown origin [7, 8].

Among these overlooked etiologies, *Toxocara canis* infection represents a relevant yet often underrecognized cause. *Toxocara* spp. are zoonotic roundworms responsible for human toxocariasis, a disease in which migrating larvae trigger a type 2 helper T-cell (Th2) immune response that frequently results in persistent eosinophilia [9–11]. Because larvae do not mature or produce eggs in humans, direct parasitological detection is not feasible; thus, serological assays particularly enzyme-linked immunosorbent assays (ELISAs) and western blots, are the mainstays of diagnosis [9, 12–14].

Several studies have shown that a substantial proportion of patients with eosinophilia of unknown origin, particularly immigrants from endemic regions, are seropositive for *Toxocara* antibodies, with the reported prevalence reaching 68% in some cohorts [9]. *T. canis* infection has been linked to eosinophilia of varying severity and may involve multiple organ systems, including the liver, lungs, and in rare cases, the pleura or central nervous system, underscoring its clinical relevance [9–11, 14, 15]. The associations among *Toxocara* seropositivity, elevated eosinophil counts, and increased total IgE have been consistently documented across diverse populations, further supporting the need to include this parasite in the differential diagnosis of unexplained eosinophilia [16, 17].

The main objective of this study was to identify undiagnosed *T. canis* infections in migrant and traveler patients from tropical and subtropical regions with eosinophilia of unknown etiology.

## Methods

This study included patients with peripheral eosinophilia who were evaluated at the Tropical Medicine Unit (TMU) of the Complejo Asistencial Universitario de Salamanca (CAUSA), Spain, between 2008 and 2023. Eligible participants met the following criteria: (i) immigration or travel from tropical or subtropical regions, (ii) availability of clinical records and microbiological data, and (iii) complete blood test results. Demographic, clinical, and laboratory data were retrospectively retrieved from medical records.

Absolute eosinophilia was defined as a peripheral blood eosinophil count ≥ 450 cells/µL. Severity was categorized as mild (450–999 cells/µL), moderate (1000–2999 cells/µL), or severe (≥ 3000 cells/µL). Relative eosinophilia was defined as a peripheral blood eosinophil percentage > 5% with a total eosinophil count < 450 cells/µL. Hyper-IgE was defined as a total serum IgE concentration > 100 U/mL.

All patients were first assessed to exclude noninfectious causes of eosinophilia, including allergic reactions, medication use, autoimmune diseases, and malignancies. A diagnostic protocol (Additional file 1: Fig. S1) was then applied to identify infectious causes through microbiological, direct, and indirect parasitic diagnostic methods, including serial stool examinations (three samples), Knott concentration technique, and serological assays for *Strongyloides*, filariae, and *Schistosoma* spp. Despite this diagnostic approach, low-burden parasitic co-infections may have remained undetected and could have contributed to eosinophilia or affected *Toxocara* serology results.

Serum samples from patients without confirmed parasitic or microbiological diagnoses were screened for anti-*Toxocara* IgG antibodies via a commercial ELISA kit (IBL International GmbH, Hamburg, Germany) according to the manufacturer’s instructions with a reported diagnostic sensitivity of 96.9% and specificity of 98.6%. The cutoff values were defined as > 11 U for positive results, 9–11 U as equivocal, and < 9 U as negative. Briefly, serum samples were diluted 1:100 in sample buffer and incubated in antigen-coated wells at 37 °C for 60 min. After washing, a horseradish peroxidase (HRP)-conjugated secondary antibody diluted 1:100 was added, followed by incubation for 30 min at 37 °C. Bound complexes were visualized via the use of tetramethylbenzidine (TMB) as the chromogenic substrate. The reaction was stopped with 0.5 M sulfuric acid, and the optical density was measured at 450 nm via a microplate reader. The results were interpreted according to the cutoff values provided by the manufacturer.

In addition, seropositive samples were subjected to Western blot (WB) analysis via a commercial kit (LDBIO Diagnostic, Lyon, France; *Toxocara* Western Blot IgG, Cat. No. TCWB.CE). Briefly, patient sera were diluted 1:100 in sample buffer and incubated with nitrocellulose strips coated with *Toxocara* excretory–secretory antigens at room temperature for 90 min. After several washes, an alkaline phosphatase–conjugated anti-human IgG secondary antibody was added, and the samples were incubated for 60 min at room temperature. The bound complexes were visualized via the use of nitro-blue tetrazolium chloride/5-bromo-4-chloro-3-indolyl phosphate (NBT/BCIP) as the chromogenic substrate. A test was considered positive if two or more bands corresponding to low-molecular-weight antigens (24–35 kDa) were observed, in accordance with the manufacturer’s diagnostic criteria.

Patient characteristics were summarized via standard measures for categorical and continuous variables. Categorical data are presented as counts and percentages, whereas continuous data are expressed as the means ± standard deviations (SDs). Univariate associations between the final diagnosis of *Toxocara canis* infection and epidemiological or clinical variables (age, sex, geographic origin, travel status, eosinophil count, total IgE, and presence of symptoms) were assessed using odds ratios (ORs) with 95% confidence intervals (CIs). *Toxocara*-seronegative individuals and the reference category of each variable were used as control groups. Fisher’s exact or *χ*^2^ tests were applied, as appropriate. Multivariate analysis was not performed due to limited sample size. Statistical significance was defined as *P* < 0.05. Analyses were conducted via SPSS version 26.0.

This study was approved by the Institutional Ethics Committee of CAUSA (reference: CEIm PI 2023/10 1448). All patient data were anonymized via unique identification codes. The study was conducted in accordance with the ethical principles of the Declaration of Helsinki, as revised in 2024 [18].

## Results

### Patient selection and eosinophilia

Of the 943 patients who attended the TMU and met the inclusion criteria, 529 (56.1%) did not have eosinophilia and were therefore excluded. Of the remaining 414 (43.9%) patients with peripheral eosinophilia, 25 (6.0%) were identified with noninfectious causes of eosinophilia, including asthma (*n* = 9), allergic rhinitis (*n* = 5), human immunodeficiency virus (HIV) infection (*n* = 2), syphilis (*n* = 2), neoplasia (*n* = 2), human T-cell lymphotropic virus type 1 (HTLV-1) infection (*n* = 1), post-transplant status (*n* = 1), Crohn’s disease (*n* = 1), Sjögren’s syndrome (*n* = 1), and rheumatoid arthritis (*n* = 1). None of the patients had received medications known to induce eosinophilia.

**Figure 1:**
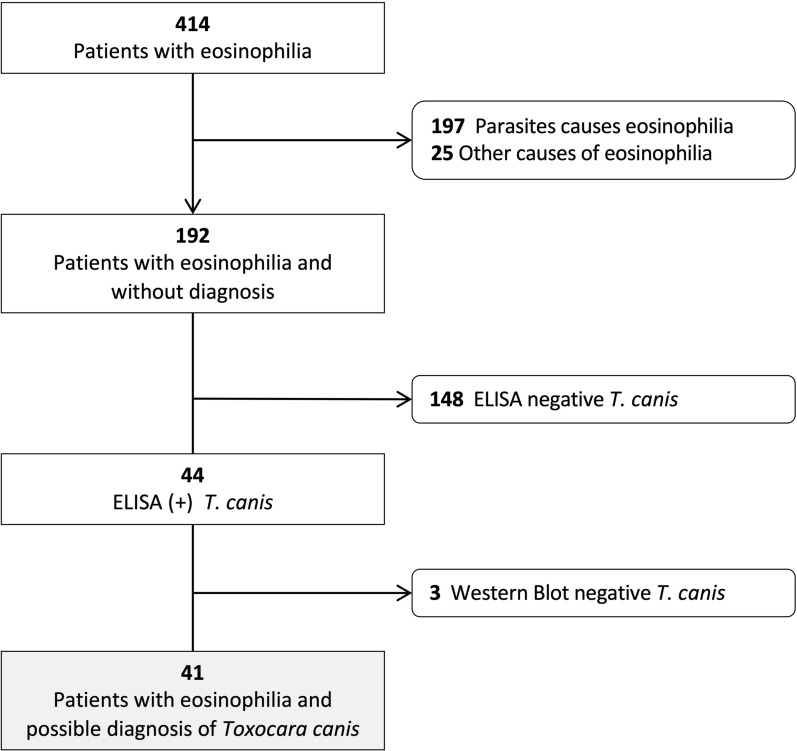
Flow diagram of patient selection

After excluding patients with noninfectious causes, the final study population consisted of 389 of 943 patients (41.2%) with eosinophilia potentially due to infectious causes. These patients were further classified according to eosinophil count: 193 of 389 patients (49.6%) had relative eosinophilia and 196 (50.4%) had absolute eosinophilia. Among this group, a parasitic infection was confirmed in 197 patients, with filariasis being the most frequent, primarily among patients from Africa (*n* = 70; 79.5%), followed by strongyloidiasis (*n* = 58; 66.7%) and schistosomiasis (*n* = 37; 84.1%). In patients from Latin America, strongyloidiasis was the most common diagnosis (*n* = 23; 26.4%). Figure 1 illustrates the stepwise patient selection process.

### Toxocara serology and WB

Of the 414 patients with eosinophilia, 192 (46.4%) did not have a confirmed microbiological or parasitological diagnosis. Serum samples from these patients were subsequently tested for *Toxocara canis* infection using ELISA for anti-*Toxocara* IgG antibodies, with positive results confirmed by western blot (WB). A total of 41 samples (21.3%) tested positive by both ELISA and WB, whereas three samples that were ELISA-positive were negative by WB (Additional file 1: Fig. S2). These three ELISA-positive but WB-negative samples were considered indeterminate and were not classified as confirmed toxocariasis, given the higher specificity of the western blot. They were therefore excluded from the subgroup used for clinical, epidemiological, and the laboratory description and from any inferential analyses.

### Demographics and epidemiology of patients positive for *Toxocara*

The majority of seropositive individuals were immigrants (*n* = 37; 90.2%), while the remaining four were travelers. The mean age was 28 ± 16.6 years (range 5–72 years), and 26 individuals (63.4%) were female. In total, 21 participants (51.2%) originated from Latin America, and 20 (48.8%) from African countries. Most resided in urban areas (61%), and 26 (66.4%) reported contact with animals, primarily dogs or/and cats. Univariate analysis comparing the 41 patients positive for *Toxocara* with 148 ELISA-negative controls demonstrated that eosinophilia < 1000 cells/μL was significantly associated with *Toxocara* seropositivity (OR 3.2, 95% CI 1.3–7.6; *P* = 0.009). No significant associations were observed for age, geographic origin, animal contact, or sex (Additional file 1: Table S1).

### Clinical and laboratory findings

The median absolute eosinophil count among ELISA-positive patients was 946.4 ± 909 cells/μL (range 153–3900 cells/μL), and the median total IgE level was 473 ± 560 U/mL (range 77.5–3078 U/mL). Of the 41 patients, 29 (70.7%) exhibited absolute eosinophilia, including 16 with mild, 11 with moderate, and 2 with severe eosinophilia. The remaining 12 patients (29.3%) had relative eosinophilia. The clinical and epidemiological characteristics of the seropositive individuals are summarized in Table 1.

**Table 1:** Epidemiological and clinical data of study participants

	*Toxocara* ELISA and WB positive patients (*n* = 41)	Percentage (%)
Demographic data		
Age (years)		
Mean ± SD	28	± 16.5
< 18 years old	15	36.6
> 18 years old	26	63.4
Sex		
Female	26	63.4
Male	15	36.6
Origin, *n* (%)		
Africa	21	51.2
Latin America	20	48.8
Type of patient		
Immigrant	37	90.2
Traveler	4	9.8
Setting		
Urban	25	61.0
Rural	9	22.0
Mixed	7	17.1
Contact with animals		
Yes	26	63.4
No	15	36.6
Clinical data		
Presence of any symptom		
Yes	24	58.5
No	17	41.5
Symptoms		
Gastrointestinal	10	41.7
Cutaneous	9	37.5
Fever	3	12.5
Others	2	8.3
Laboratory data		
Count eosinophils (cell/μL), mean ± SD	946.4	± 909
Count IgE, mean ± SD	473	± 560.8

*IgE* immunoglobulin E, SD ± standard error

**Figure 2:**
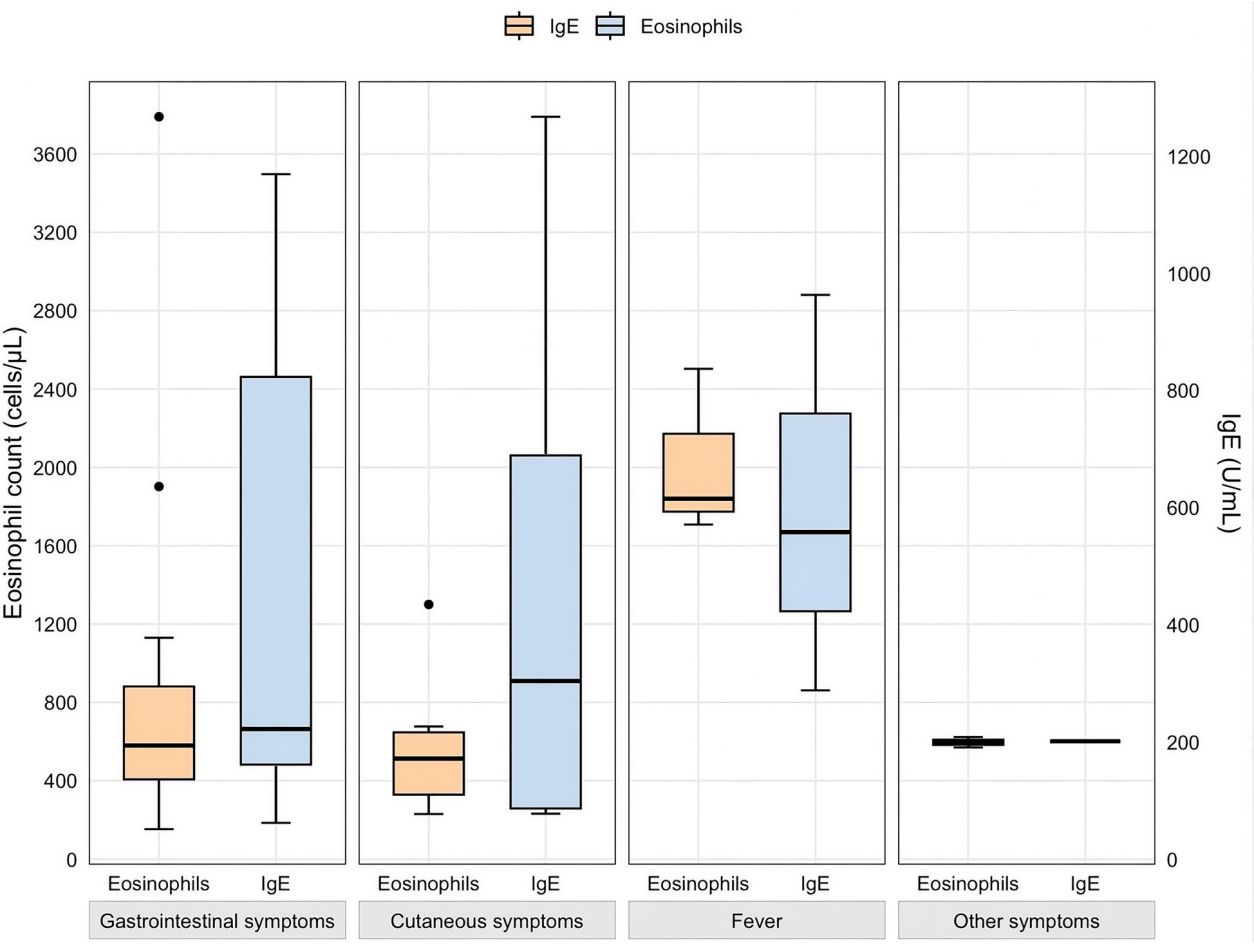
Relationships between eosinophil count, immunoglobulin E (IgE) level, and symptoms

Clinical manifestations were variable: abdominal complaints were reported in 10 patients (24.4%), pruritus in 9 (22%), fever in 3 (7.3%), and other symptoms, such as headache or eye discomfort, in 2 patients (4.9%); notably, 17 patients (41.5%) were asymptomatic. The detailed clinical features of these 41 patients are summarized in Table 2. The intensity of eosinophilia and IgE levels correlated with clinical manifestations, with patients presenting with fever exhibiting higher eosinophil counts and IgE levels compared with those with gastrointestinal, cutaneous, or other symptoms (Fig. 2).

**Table 2:** Epidemiological, clinical, laboratory, and outcome features of patients diagnosed positive for *T. canis*

Case no.	Initial protocol	Age, year/sex	Origin or travel destination	Setting	Contact with animals _a_	Symptoms at presentation	IgE	Absolute eosinophil count	(% of WBC)
1	T	45/M	Ethiopia	Mixed	Yes	Diarrhea	188	153	6
2	I	34/F	Colombia	Urban	Yes	Memory loss	–	570	8.5
3	I	38/M	Bolivia	Rural	Yes	None	–	350	5.2
4	I	5/F	Equatorial Guinea	Rural	Yes	None	590	1530	19.5
5	I	14/M	Nigeria	Urban	Yes	None	118	292	6.26
6	I	13/F	Equatorial Guinea	Urban	Yes	None	308	600	1.6
7	I	18/F	Equatorial Guinea	Rural	Yes	Pruritus, abdominal pain, headaches, eye discomfort	304	230	7.1
8	I	17/F	Equatorial Guinea	Urban	No	Hand lesions, cough	1267	329	5.37
9	I	14/M	Equatorial Guinea	Urban	Yes	Occasional vomiting, headaches	1012	580	11
10	I	15/F	Equatorial Guinea	Urban	No	None	123	180	5.7
11	I	17/F	Equatorial Guinea	Urban	Yes	Pruritus, abdominal pain	827	429	5.09
12	I	18/M	Equatorial Guinea	Urban	No	None	3078	3900	40.4
13	I	18/M	Senegal	Mixed	Yes	None	330	350	10.3
14	I	43/M	Colombia	Rural	Yes	Digestive discomfort	172	633	7
15	I	18/M	Dominican Republic	Rural	Yes	Abdominal pain, headaches	256	364	5.48
16	I	21/M	Equatorial Guinea	Urban	No	Eye discomfort	201	623	8.49
17	I	17/F	Dominican Republic	Urban	No	None	116	1290	14.8
18	I	8/F	Equatorial Guinea	Urban	No	Epigastric pain	129	517	11
19	I	12/M	Equatorial Guinea	Urban	No	Pruritus, cough	79.4	677	5.55
20	I	12/F	Equatorial Guinea	Urban	No	None	130	499	6.27
21	I	12/F	Equatorial Guinea	Urban	No	Epigastric pain	760	593	6.29
22	I	9/F	Equatorial Guinea	Rural	Yes	Pruritus, eye discomfort	198	526	9.79
23	I	18/M	Equatorial Guinea	Urban	No	Pruritus, headaches	86,4	250	5.91
24	I	21/M	Equatorial Guinea	Urban	Yes	Pruritus	405	647	7.47
25	I	31/F	Brazil	Mixed	No	Epigastric pain	–	1130	9
26	I	28/F	Bolivia	Urban	Yes	Pruritus	77.5	513	10.1
27	I	42/M	Bolivia	Mixed	Yes	Fever	558	2503	22
28	T	42/F	Equatorial Guinea	Urban	No	Fever	963	1840	3.1
29	I	30/F	Nigeria	Urban	No	Diarrhea, pruritus	–	3790	37
30	I	58/M	Bolivia	Urban	Yes	None	505	160	6
31	I	36/M	Bolivia	Rural	Yes	None	81	1504	20
32	I	41/F	Bolivia	Urban	Yes	None	146	480	2
33	I	8/F	Bolivia	Rural	Yes	None	502	2670	35
34	T	50/F	Bolivia	Mixed	Yes	Fever	288	1708	17
35	I	59/F	Bolivia	Urban	Yes	None	403	610	1.6
36	I	43/F	Paraguay	Urban	Yes	None	165	500	51
37	I	47/F	Bolivia	Urban	Yes	Digestive discomfort	1169	1902	33
38	I	40/F	Honduras	Mixed	No	None	210	506	7
39	I	28/F	Colombia	Urban	No	Pruritus	689	1300	15
40	I	17/M	Dominican Republic	Mixed	Yes	Pruritus, cough	–	1160	10.6
41	I	52/F	Brazil	Urban	Yes	None	528	728	2.4

*I* immigrants, *T* traveler, *M* male, *F* female, *IgE* immunoglobulin E, *WBC* white blood cell, *WB* western blot

^a^Contact with animals refers specifically to exposure to dogs and/or cats

## Discussion

Eosinophilia in immigrant and traveler populations represents a diagnostic challenge in clinical practice, as reflected by our findings, due to the wide range of potential causes, nonspecific clinical symptoms, and the frequent presence of mild or asymptomatic presentations. This diagnostic gap is driven by limitations in routine diagnostic methods, including the limited sensitivity of standard fecal microscopy for many helminths [1, 7, 19], as well as incomplete consideration of the differential diagnosis. Consequently, less frequently recognized etiologies, such as *Toxocara canis*, may be overlooked despite their known prevalence in tropical and subtropical regions [20, 21]. In our cohort, nearly one-quarter of patients with unexplained eosinophilia tested positive for *Toxocara canis* by ELISA with a seropositivity rate of 23%, consistent with previous reports ranging from 5.5% to 68% [16, 22–24], reflecting geographic and population differences.

The high concordance between ELISA and western blot (> 93%) supports the reliability of ELISA as a screening tool, with western blot reserved for cases requiring additional diagnostic certainty. While these assays are highly sensitive and specific for *Toxocara canis*, cross-reactivity with *Toxocara cati* or other nematodes cannot be entirely excluded due to antigenic similarities. Techniques such as serum pre-absorption have been proposed to reduce nonspecific reactivity [25]. In our study, this limitation was mitigated through a structured diagnostic protocol that systematically excluded other parasitic infections. Therefore, serological results should be interpreted cautiously and within the appropriate clinical and epidemiological context, highlighting the need for more specific diagnostic tools for toxocariasis.

In our study, *T. canis* seropositivity was significantly associated with eosinophil counts below 1000 cells/μL. Although toxocariasis has traditionally been linked to marked eosinophilia [9, 26], growing evidence indicates that many infections present with only mild-to-moderate eosinophilia or even normal counts, particularly in adults [10, 29]. This variability has also been observed in cohort studies, where toxocariasis is a frequent cause of unexplained eosinophilia, often in asymptomatic or minimally symptomatic individuals, with eosinophil levels below the threshold for hypereosinophilia (> 1500/μL) [9, 10], consistent with a pattern of moderate eosinophil elevations rather than extreme counts [27, 28].

The demographic profile of our patients, predominantly immigrants and travelers from Africa and Latin America; aligns with existing evidence linking *Toxocara* infection to regions with high environmental exposure [31, 32]. The predominance of nonspecific symptoms, such as abdominal complaints and pruritus, combined with a high proportion of asymptomatic cases, underscores the diagnostic challenges of this parasitic infection. Notably, among patients with confirmed toxocariasis, those presenting with fever presented significantly higher eosinophil counts and IgE levels, suggesting that fever may reflect a more robust systemic inflammatory response, potentially associated with more intense larval migration [12, 13].

This immunological pattern is further supported by the overall profile, characterized by absolute eosinophilia and elevated total IgE, consistent with a T helper 2 cell (Th2)-polarized immune response [13]. The pathophysiology of eosinophilia in toxocariasis is mediated by this Th2 response to migrating larvae, which stimulates increased eosinophil production and activation. However, the magnitude of eosinophilia is highly variable and depends on host-related factors, parasite burden, and the extent of tissue or organ involvement [13, 30]. Milder elevations are more commonly observed in otherwise healthy individuals or in cases with limited tissue involvement, where eosinophilia may be the only laboratory abnormality [16]. Additionally, eosinophilia in toxocariasis can be transient, and in some cases, may resolve spontaneously over time, particularly when severe or progressive organ involvement is absent.

## Conclusions

*Toxocara canis* infection is a relevant and frequently underrecognized cause of eosinophilia among immigrants and travelers. Nearly a quarter of patients with unexplained eosinophilia met the serological criteria for toxocariasis, most of whom would have remained undiagnosed without routine screening. These findings underscore the importance of systematically including *Toxocara* spp. testing in the workup of eosinophilia after more common etiologies have been excluded.

## Supplementary Information

Additional file1: Figure S1: Flowchart of the diagnostic protocol for a patient attending a consultation at the Tropical Medicine Unit. Figure S2: Full-length uncropped western blot of *Toxocara* ELISA-positive sera. Table S1. Univariate associations between *Toxocara *in patients with unexplained eosinophilia.

## Data Availability

All data generated and analyzed during this study are included in this published article and its supplementary information files.

## Abbreviations

ELISA: Enzyme-linked immunosorbent assay
WB: Western blot
Th2: Type 2 helper T cell
IgG: Immunoglobulin G
IgE: Immunoglobulin E
