# Supplementary material for: Toxocariasis in immigrants and travelers with unexplained eosinophilia
Source: Parasit Vectors. 2026 Feb 20;19:133. doi: 10.1186/s13071-026-07300-9 (PMC13032616; doi:10.1186/s13071-026-07300-9)
Supplement: Supplementary file 1 — Additional file1: Figure S1: Flowchart of the diagnostic protocol for a patient attending a consultation at the Tropical Medicine Unit. Figure S2: Full-length uncropped western blot of Toxocara ELISA-positive sera. Table S1. Univariate associations between Toxocara in patients with unexplained eosinophilia. [file 13071_2026_7300_MOESM1_ESM.docx]

**Supplementary information**

Fig. S1: Flowchart of the diagnostic protocol for a patient attending a consultation at the Tropical Medicine Unit.


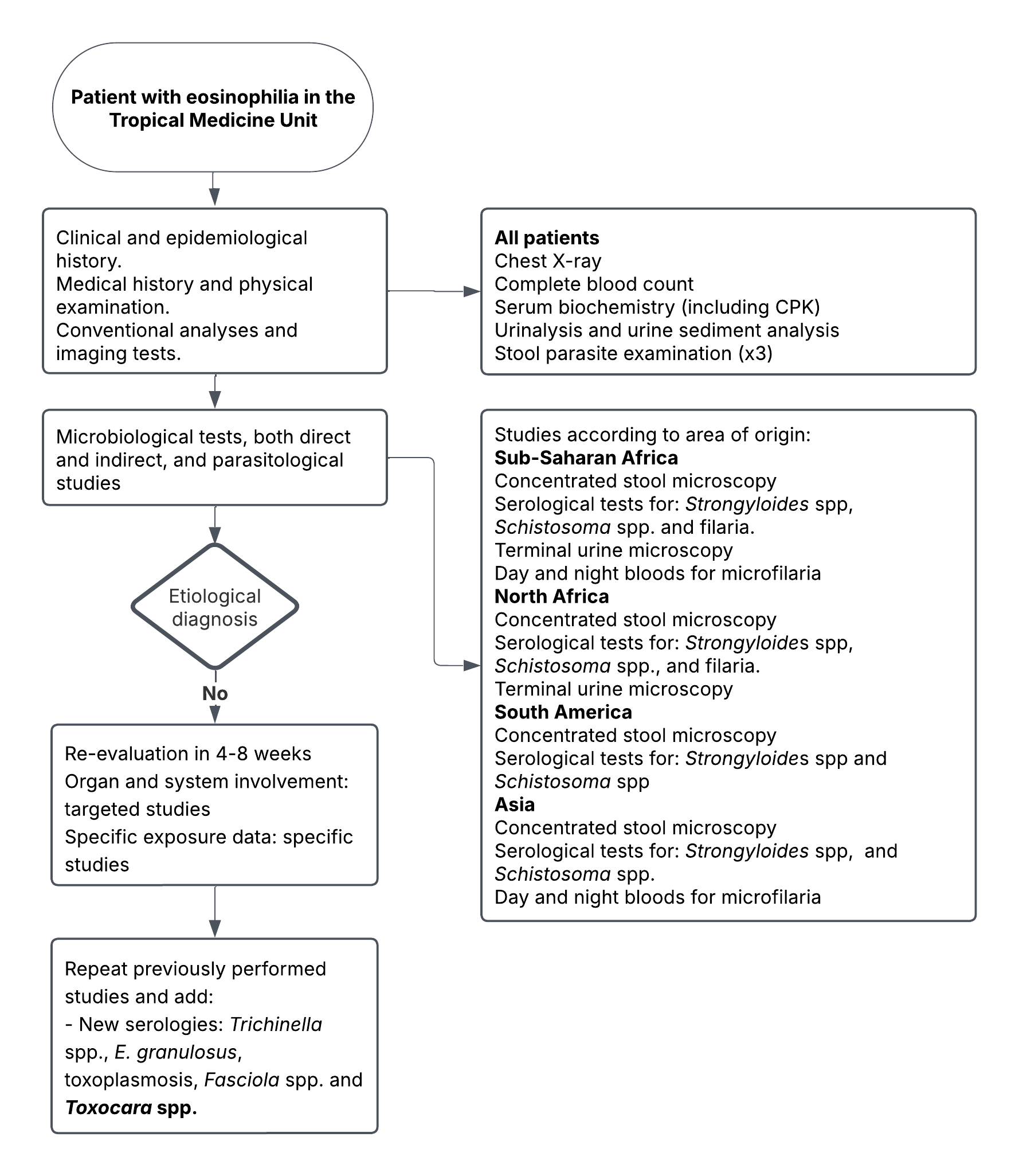


Fig. S2: Full-length uncropped Western blot of Toxocara ELISA-positive sera


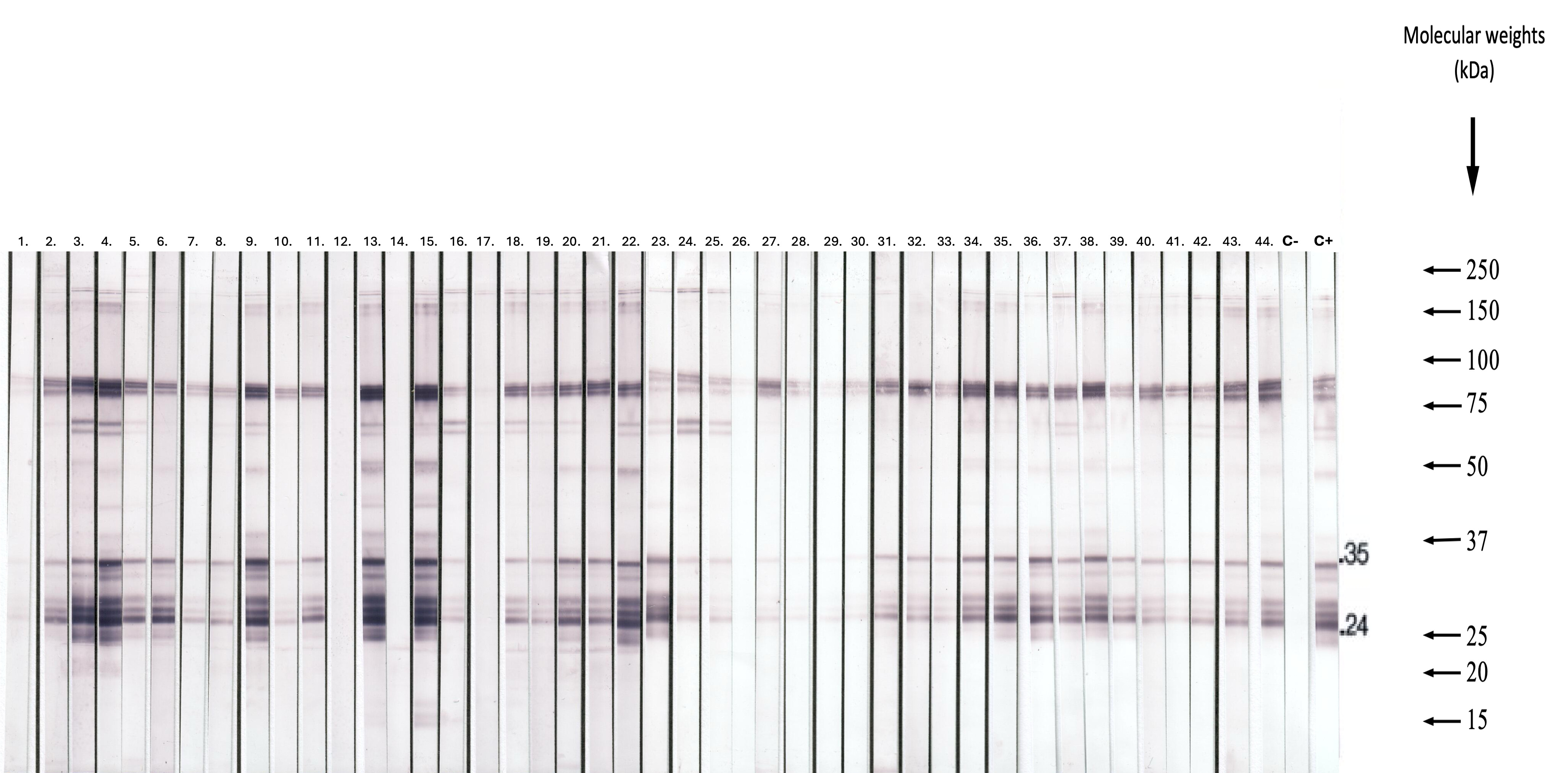


Table S1. Univariate associations between *Toxocara* in patients with unexplained eosinophilia.

| Variable | *Toxocara* ELISA and WB positive patients (n=41) | *Toxocara* ELISA and WB negative patients (n=148) | OR | 95% CI | p-value |
| --- | --- | --- | --- | --- | --- |
| Eosinophil count |  |  |  |  |  |
| <1000 cells/µL | 32/41 (78.0%) | 78/148 (52.7%) | 3.2 | 1.3–7.6 | 0.009* |
| ≥1000 cells/µL | 9/41 (22.0%) | 70/148 (47.3%) | Ref |  |  |
| Age |  |  |  |  |  |
| <18 years | 15/41 (36.6%) | 50/148 (33.8%) | 1.1 | 0.5–2.4 | 0.74 |
| ≥18 years | 26/41 (63.4%) | 98/148 (66.2%) | Ref |  |  |
| Geographic origin |  |  |  |  |  |
| African | 21/41 (51.2%) | 75/148 (50.7%) | 1.0 | 0.5–2.1 | 0.96 |
| Latin American | 20/41 (48.8%) | 73/148 (49.3%) | Ref |  |  |
| Animal contact |  |  |  |  |  |
| Yes (dogs/cats) | 26/41 (63.4%) | 90/148 (60.8%) | 1.1 | 0.5–2.4 | 0.76 |
| No | 15/41 (36.6%) | 58/148 (39.2%) | Ref |  |  |
| Sex |  |  |  |  |  |
| Female | 26/41 (63.4%) | 85/148 (57.4%) | 1.3 | 0.6–2.7 | 0.51 |
| Male | 15/41 (36.6%) | 63/148 (42.6%) | Ref |  |  |

OR= Odds ratio; CI= Confidence interval; Ref= reference category.

*Statistically significant.
